# Subchondral H-type blood vessel formation aggravates articular cartilage degeneration through LEP-LEPR axis

**DOI:** 10.3389/fmed.2026.1751127

**Published:** 2026-03-13

**Authors:** Chao Yu, Xi Zhao, Shitong Luo, Jun Qin, Jingjing Li

**Affiliations:** 1 Department of Orthopaedic Surgery, The University-Town Hospital of Chongqing Medical University, Chongqing, China; 2 Molecular Oncology Laboratory, Department of Orthopaedic Surgery and Rehabilitation Medicine, The University of Chicago Medical Center, Chicago, IL, United States; 3 Department of Oncology, School of Clinical Medicine, The Affiliated Hospital, Shandong Second Medical University, Weifang, China

**Keywords:** cartilage injury, leptin, leptin receptor (LEPR), lipid metabolism, osteoarthritis

## Abstract

**Background:**

Osteoarthritis (OA) is a chronic degenerative joint disease characterized by cartilage deterioration that has resulted in severe physical and economic costs for society. Risk factors for osteoarthritis encompass genetic predisposition, mechanical influences, obesity, inflammation, and metabolic problems. Recent reports indicate that the morphological alterations of subchondral bone evolve dynamically with the advancement of osteoarthritis. A significant alteration in subchondral bone is the development of aberrant blood vessels, particularly H-type vessels. H-type arteries are essential for preserving subchondral bone homeostasis. They support nutrients and oxygen to bone tissue and eliminate waste, thereby supporting the metabolic activities of osteoblasts and osteoclasts. However, the upstream factors governing H-type arteries are yet unknown.

**Methods:**

The whole transcriptome analysis was performed by informatics using the GSE51588 dataset, which includes subchondral bone samples from OA patients, comparing the OA lesion side with the non-lesion side, and was further verified in clinical samples. Using LEP and LEPR knockout mice, the correlation between abnormal subchondral bone metabolism and LEP, LEPR, and H-type vessel formation were uncovered.

**Conclusion:**

This study reveals that the lipid metabolism pathway was highly enriched on the OA lesion, and LEP and LEPR were markedly underexpressed in the subchondral bone on the side with severe OA lesions. The causal relationship of the LEP-LEPR-CD31 signaling pathway in aging and osteoarthritis models is revealed at the overall and molecular levels. This project will provide a new theoretical basis for the pathogenesis of early OA and new ideas and targets for the clinical treatment of OA by exploring the LEP-LEPR-CD31 signaling in osteoarthritis.

## Introduction

Osteoarthritis (OA) is a chronic, degenerative joint disorder marked by the deterioration of cartilage. It commonly manifests in weight-bearing joints and those subjected to heavy activity, serving as the primary cause of physical disability (1). As human life expectancy rises, the global health and economic ramifications of osteoarthritis will persistently escalate in the future (2). The pathological features of osteoarthritis include cartilage degradation, synovial inflammation, osteophyte development, and subchondral bone remodeling (3). Notwithstanding the presence of established risk factors such as genetic predisposition, mechanical influences, obesity, inflammation, and metabolic disorders, the precise pathophysiology remains ambiguous, leading to suboptimal treatment success in most patients. Consequently, investigating the fundamental etiology of osteoarthritis has emerged as a critical subject requiring immediate attention (4).

Recent evidence indicates that articular cartilage degradation is associated with aberrant subchondral bone remodeling (5–7). Subchondral bone offers mechanical support to articular cartilage and collaborates with it to convey pressure loads, ensure joint congruence, and avert stress concentration inside the joint. In contrast to the slower remodeling rate of articular cartilage, subchondral bone exhibits a more rapid remodeling process in reaction to alterations in the mechanical environment (8). The morphological alterations of subchondral bone evolve dynamically with the advancement of osteoarthritis. During OA progression, subchondral bone undergoes dynamic structural alterations characterized by early increased remodeling followed by late-stage sclerosis (9, 10). The mechanism that triggers aberrant subchondral bone production and the correlation between heightened subchondral bone remodeling and articular cartilage degradation in osteoarthritis remain ambiguous.

A significant alteration in osteoarthritis subchondral bone is the development of aberrant blood vessels, particularly H-type blood vessels (9). H-type blood arteries possess distinctive morphology, positioning, and functionality. They are predominantly located in subchondral bone. Their endothelial cells have elevated expression of platelet endothelial cell adhesion molecule-1 (PECAM-1/CD31) and endomucin (EMCN). H-type arteries are essential for sustaining subchondral bone homeostasis. They supply nutrients and oxygen to bone tissue and eliminate waste, therefore facilitating the metabolic functions of osteoblasts and osteoclasts (11). Besides their function in bone metabolism, H-type arteries are linked to the pathogenesis of osteoarthritis (OA) (12). Excessive vascularization, especially H-type arteries, in subchondral bone is deemed responsible for aberrant subchondral bone remodeling and cartilage deterioration in osteoarthritis (13, 14). Accumulating evidence suggests that alterations in H-type vessel density correlate with OA progression and may promote inflammatory infiltration and abnormal bone remodeling (12, 15, 16).

Leptin (LEP) is a hormone which is crucial for the regulation of energy equilibrium, hunger, and body mass. Adipose tissue produces and releases it, influencing many tissues, including bones, and positively affecting bone metabolism (17). The leptin receptor (LEPR) is a membrane protein expressed in multiple tissues, including bone, and is crucial for modulating the effects of LEP (17). Upon binding to LEPR, LEP stimulates the signaling system that governs bone metabolism, enhances the proliferation and differentiation of osteoblasts, and suppresses osteoblast death, thereby augmenting bone production (18). Furthermore, LEP decreases the development and activity of osteoclasts, hence diminishing bone resorption (19). Deficiency in LEP or LEPR can result in alterations in bone metabolism and heightened bone loss, signifying that their modulation is crucial for sustaining bone health (20). Furthermore, LEP and LEPR participate in the regulation of lipid metabolism. Deficiencies in LEP and LEPR might result in lipid metabolism abnormalities, augmented adipose tissue bulk, and elevated levels of free fatty acids (FFAs), potentially causing excessive adipocyte growth and chronic inflammation in adipose tissue (21). Elevated concentrations of free fatty acids (FFAs) might catalyze the angiogenesis process by enhancing the expression of vascular endothelial growth factor (VEGF), so facilitating the proliferation and differentiation of endothelial cells (22, 23). Obesity and dysregulated fatty acid metabolism are significant risk factors for osteoarthritis. Therefore, we hypothesized that the LEP–LEPR axis may influence the development of subchondral H-type vessels through modulation of lipid metabolism.

This study initially established the association between defective subchondral bone metabolism and LEP, LEPR, and H-type blood vessel development, based on LEP and LEPR knockout mice. The causal link of the LEP-LEPR-CD31 signaling pathway in aging and osteoarthritis models is elucidated. Our goal aims to establish a novel theoretical framework for the pathophysiology of early osteoarthritis and to propose innovative concepts and targets for its therapeutic therapy by investigating the LEP-LEPR-CD31 signaling pathway in osteoarthritis.

In this manuscript writing, we followed the guideline suggest by Transparency In The reporting of Artificial INtelligence – the TITAN guideline (24).

## Methods and materials

### Animal assay

Experimental mice including wild-type (WILD TYPE-Leprem2Cd479/Gpt), diabetic (db/db) (WILD TYPE-Leprem2Cd479/Gpt), and obese (ob/ob) (B6/JGpt-Lepem1Cd25/Gp) mice, aged 6–7 weeks, were acquired from Jiangsu Jicui Yaokang Biotechnology Co., Ltd. and maintained in the SPF-level laboratory of the Experimental Animal Center at Chongqing Medical University. The mice were maintained at a constant temperature of 22 ± 2 °C, relative humidity of 55 ± 5%, and a light–dark cycle of 12 h. Throughout the breeding procedure, mice were ensured unrestricted access to water and food, and all activities were conducted in compliance with the protocol sanctioned by the Animal Ethics and Use Committee of Chongqing Medical University (No. 2021.5.28/LL-202133). For experimental procedures, mice were anesthetized with isoflurane inhalation (induction at 3–4% and maintenance at 1.5–2% in oxygen). At the study endpoint, mice were euthanized by CO₂ inhalation using a gradual fill method with a displacement rate of 30% of the chamber volume per minute, followed by cervical dislocation to ensure death. This work has been reported in accordance with the ARRIVE guidelines (Animals in Research: Reporting *In Vivo* Experiments) (25).

### Data source and preprocessing

The GSE51588 gene expression profile dataset was obtained from the GEO database, comprising 40 subchondral bone samples from human OA tibial plateaus (20 lateral tibial plateaus [LT] vs. 20 medial tibial plateaus [MT]) from *Homo sapiens*. The dataset was annotated to convert probes to gene symbols, and standardized using R (version 4.1.3, www.R-project.org) and the core preprocessing module in GEO2R.

### Whole-transcriptome informatics analysis

Differentially expressed genes (DEGs) between OA lesion (LT) and non-lesion (MT) sides were identified using standard thresholds (|log2 fold change| > 1 and *p* < 0.05). Gene Ontology (GO) and Kyoto Encyclopedia of Genes and Genomes (KEGG) enrichment analyses were conducted using R packages, and Gene Set Enrichment Analysis (GSEA) was performed to identify significantly enriched pathways. *p* values <0.05 were considered statistically significant. All analyses were performed using R (version 4.1.3).

### Specimen processing

Human sample collection was approved by the Ethics and Committee of Chongqing Medical University (2023-021). Human tissue specimens and murine knee joints were fixed in 4% paraformaldehyde for 36–48 h, decalcified using 20% ethylenediaminetetraacetic acid (EDTA with pH 7.4) for 21 days, embedded in paraffin, and sagittal slices of the murine joints were prepared at a thickness of 7 μm. The samples were stained with Safranin O-fast green, Toluidine blue, Sirius red, Masson, TRAP, and Alcian blue, among others, and subjected to immunohistochemistry and immunofluorescence techniques.

### Toluidine blue staining

Slides were produced as described above, thereafter undergoing dewaxing and hydration. The slides were thereafter submerged in water briefly and incubated in a 0.25% toluidine blue dye solution for 2 to 5 min. Subsequently, excess dye solution was eliminated with water; the slices were rinsed with water and subsequently examined under a microscope using 0.1% glacial acetic acid. Cartilage and mast cells have distinct coloration, whereas the backdrop transitions to a light blue tint. Rinsing with tap water to cease differentiation, followed by drying with air. The slides were further dehydrated with anhydrous ethanol, rendered translucent with xylene, and then encapsulated in neutral gum. Toluidine blue can provide a purple-red coloration to chondroitin sulfate in cartilage tissue. This technique is utilized to investigate abnormal changes and the distribution of mast cells, subsequently analyzing the morphological structure of cartilage.

### TRAP staining

A TRAP working solution was formulated by sequentially incorporating 50 μL of para-fuchsin solution (G1050-2), 50 μL of sodium nitrite solution (G1050-3), 100 μL of AS-BI phosphate substrate solution (G1050-4), and 1.8 mL of reaction buffer (G1050-1) into a centrifuge tube. Meticulously combine at each phase, thereafter filter using a 0.45 μm needle filter membrane, followed by dewaxing the paraffin sections and rinsing with distilled water for several minutes. The slides were subsequently outlined using a histochemical pen and positioned in a humidified chamber. An appropriate volume of distilled water was added and incubated at 37 °C for 2 h. After the removal of water, the pre-prepared TRAP working solution was enough applied to cover the slides and incubated at 37 °C in darkness for 20–30 min. Subsequently, the slides underwent the standard dehydration and mounting procedures. TRAP staining in osteoclasts displays a wine-red hue and is located within the cytoplasm.

### Masson staining

In accordance with standard protocol, dewax the paraffin sections, drain them, and immerse them in solution A overnight; Following a 30 min incubation at 65 °C, rinse the sections with tap water the subsequent day to eliminate any residual solution A; Equal volumes of solutions B and C must be amalgamated, followed by a one-minute staining period and a subsequent fifteen-second washing with water. Differentiate for one to two minutes using a 1% hydrochloric acid alcohol solution (concentrated hydrochloric acid: anhydrous ethanol in a 1:100 ratio). Note: that the cell nucleus appears gray-black, but the backdrop is nearly colorless or light gray. Rinse with water and expel excess moisture to prevent differentiation. The specimen must be immersed in solution D for 6 to 8 min, ceasing when the tissue exhibits a vivid red coloration. Rinse with water to eliminate any excess liquid, then immerse in solution E for approximately 1–2 min. Note that while the other fibers are red, the collagen fibers exhibit a mild red hue. Rinse three times for approximately eight seconds each with 1% glacial acetic acid after immersion in F solution for two to thirty seconds. Employ anhydrous ethanol for dehydration for approximately five, ten, and thirty seconds. After 30 min and 2 min of n-butanol dehydration, clarify the slide with xylene and affix it using neutral gum. Following Masson’s trichrome staining, collagen fibers exhibit a spectrum from sky blue to deep blue, red blood cells appear light red, while cellulose, muscle fibers, cytoplasm, and keratin display hues ranging from red to purplish red.

### Sirius red staining

Slides were produced as described above. Slides were immersed in Sirius red complex staining solution for 5–10 min. Then rinsing the slides with water, dehydrating using n-butanol for 30 s and 2 min, rendering transparent with xylene, and mounting the slide with neutral glue.

Collagen fibers in tissues are dyed red under a standard optical microscope. Observation using a polarized light microscope helps differentiate among distinct fiber kinds.

### Alcian blue staining

Slides were produced as described above. The slides were immersed in Alcian blue dye solution for 15–30 min and rinsed with tap water. Submerge parts in nuclear quick red dye solution for 3 min and rinse with tap water. Dehydrate sections using anhydrous ethanol at three intervals of five minutes, clarify with xylene for two intervals of five minutes, and mount with neutral gum. Results Interpretation: Alcian blue is a particular stain for acidic mucins, rendering acidic mucopolysaccharides in the cytoplasm blue and neutral glycoproteins red, thus differentiating acidic glycoproteins from neutral glycoproteins.

### H-type vessel localization and quantification

To specifically identify H-type vessels in subchondral bone, immunofluorescence double staining was performed using antibodies against CD31 (PECAM-1) and EMCN. Sections of the subchondral bone beneath the medial and lateral tibial plateaus were selected for analysis. H-type vessels were defined as CD31^high/EMCN^high microvessels. Vessel density (number of H-type vessels per mm^2^) and vessel area fraction were quantified using ImageJ software. At least three non-overlapping fields per section and three sections per sample were analyzed to ensure representative quantification. This procedure allows clear distinction of H-type vessels from other microvessels with lower CD31 or EMCN expression.

### Statistical analyses

All experiments were independently conducted at least three times or performed across three separate experimental batches. Statistical analyses were carried out using GraphPad Prism 7, and the results are presented as mean ± standard deviation (SD). Group comparisons were evaluated using one-way ANOVA or Student’s *t*-test where appropriate. A *p*-value <0.05 was considered indicative of statistical significance.

## Results

### LEP and LEPR are associated with subchondral bone pathological changes in OA

**Figure 1:**
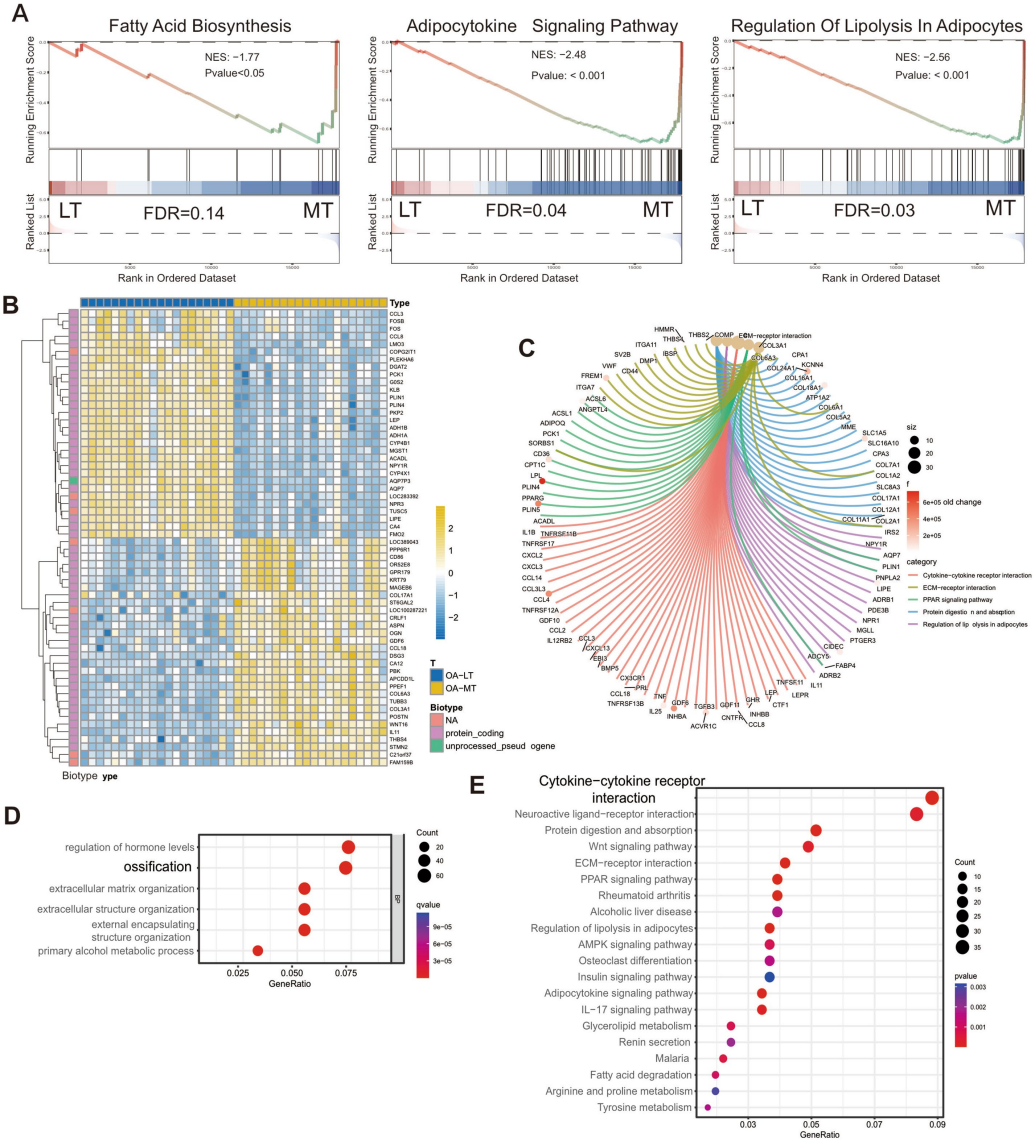
**(A)** GSEA analysis of gene expression profile of medial subchondral bone (MT) and lateral subchondral bone (LT) in OA. **(B)** Heat map of DEGs. **(C)** Peacock map display of the important DEGs genes in the KEGG pathway. **(D)** GO analysis of DEGs. **(E)** KEGG pathway enrichment analysis of DEGs.

Clinically, patients with medial osteoarthritis classified grade 3–4 as Kellgren-Lawrence demonstrate considerable deterioration of the medial tibial plateau cartilage and subchondral bone sclerosis, while the lateral tibial plateau cartilage is predominantly intact, and the subchondral bone architecture is typically normal. To examine the relationship between the pathological characteristics of the medial subchondral bone and changes in the gene expression profile of the subchondral bone, we analyzed the RNA sequencing data from GEO database (GSE51588), which includes the comprehensive genome expression profile chip data of the medial tibial plateaus (MT) and lateral tibial plateaus (LT) of the subchondral bone from 20 individuals with medial knee OA. We used the data from the LT of OA subchondral bone as the control group and MT as the experimental group, GSEA analysis was performed based on the recorded 23,002 gene expression data points. The results show that unlike the lateral subchondral bone, the fatty acid production, adipocytokine signaling pathway, and lipolysis regulation in the adipocytes of the medial subchondral bone were significantly inhibited (Figures 1A,C).

To further explore the relationship between subchondral bone pathological changes and gene regulation, we used the GEO2R preprocessing core package of the GEO database to screen 23,002 genes in the dataset for differentially expressed genes (*p* < 0.05) and obtained a total of 11,181 differentially expressed genes (DEGs) (Supplementary Figure 1A).

Then, we performed Gene ontology (GO) analysis and Kyoto Encyclopedia of Genes and Genomes (KEGG) analysis based on DEGs (Figure 1D). Enriched biological process (BP) analysis shows that regulation of hormone levels and ossification terms are significantly enriched (Figure 1E). Besides, molecular function (MF) analysis suggests that receptor-ligand activity and signaling receptor activator activity were significantly enriched, collagen-containing extracellular matrix on cell components (CC) was significantly enriched, and cytokine-cytokine receptor interaction in KEGG pathway was significantly enriched.

To find the key genes regulating subchondral bone lesions, we further screened the genes with expression differences of more than 2 times and converted the fold change (FC) values by taking the logarithm (log2) to further narrow the range of differentially expressed genes. We obtained 1,005 significantly differentially expressed genes (*p* < 0.05, log2≧1 or ≦ − 1), of which 422 genes were up-regulated, and 583 genes were down-regulated. The significantly differentially expressed genes were then constructed using the String protein database^1^ to construct a PPI network, and hub gene screening was performed using Cytoscape, obtaining 44 hubs such as LIPE, FABP4, LPL, LEP, and LEPR. Gene (Supplementary Figures 1B–D), combined with the results of GSEA, GO, and KEGG analysis, and combined with the heat map significantly different gene list (Figure 1B), it was found that LEP, LEPR molecules and signaling pathways can simultaneously meet the results of the above bioinformatics analysis. Therefore, we assume that LEP and LEPR may be related to abnormal lipid metabolism and pathological changes in subchondral bone.

**Figure 2:**
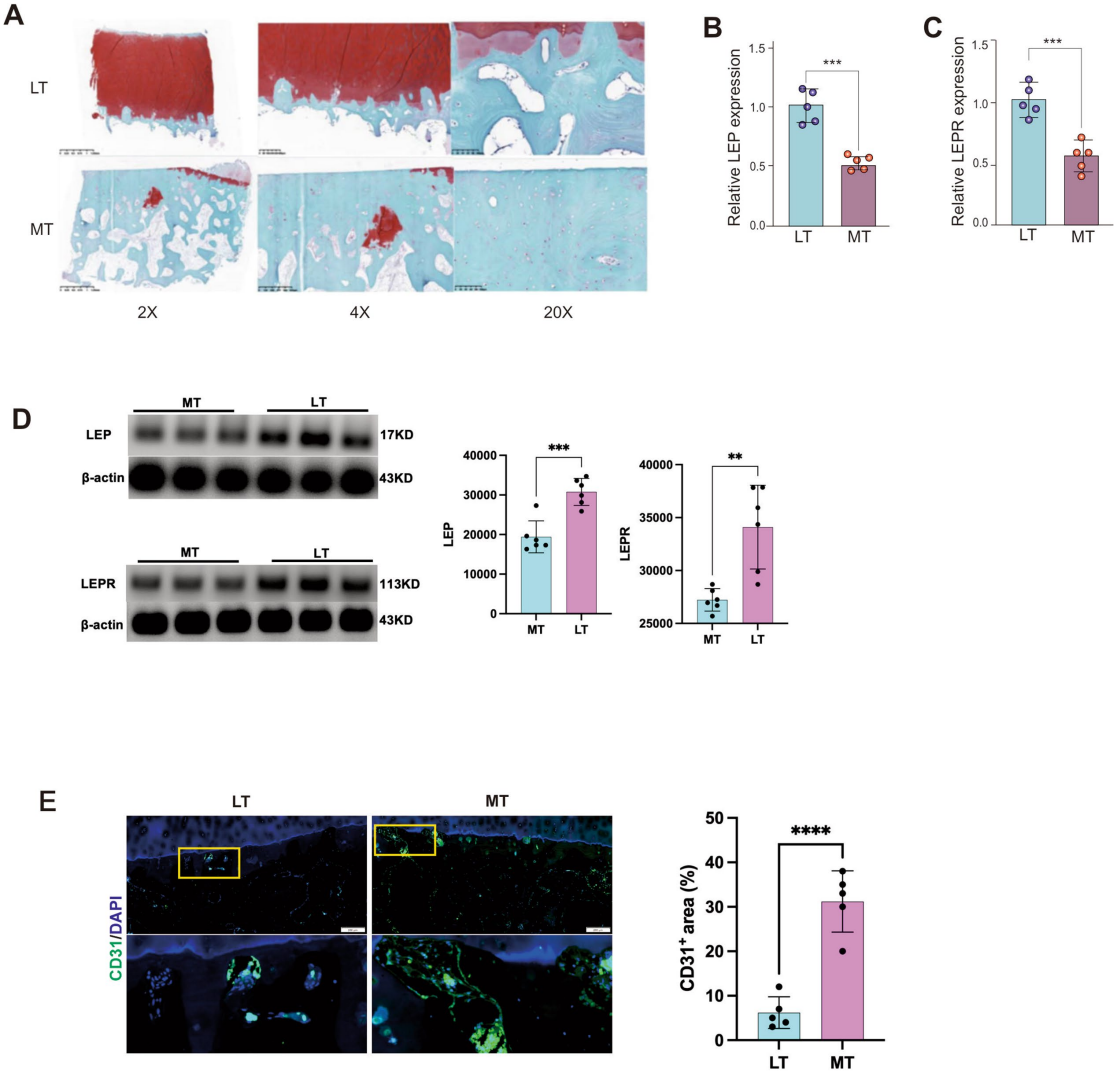
**(A)** Representative safranin O/fast green stain in samples from MT and LT subchondral bone of OA patients. **(B,C)** PCR detection of the expression of LEP and LEPR in MT and LT subchondral bone of OA patients. **(D)** Representative western blot images (left) and corresponding quantitative analysis (right) of LEP and LEPR protein levels in MT and LT subchondral bone of OA patients. **(E)** Representative immunofluorescence images (left) showing CD31 (green) expression in the subchondral bone area; nuclei are counterstained with DAPI (blue). High-magnification views of the boxed areas are shown below. Data are expressed as mean ± SD (*n* = 6 per group).* Indicates *p* value <0.05, ** indicates *p* value<0.01, *** indicates *p* value <0.001,**** indicates *p* value <0.0001.

To verify the results of bioinformatics analysis, we selected 5 human medial OA specimens (K-L grade 3–4) collected in the early stage. Through scanning observation after Safranin O-Fast Green staining, compared with the lateral tibial plateau, the cartilage defect on the medial lesion side was serious and the defect area was large. Subchondral bone sclerosis was seen, and many cell cavities were seen inside (Figure 2A). At the same time, we extracted RNA from the medial and lateral subchondral bone tissues, respectively, and performed PCR detection of LEP and LEPR genes. Consistently, it was found that the expression levels of LEP and LEPR in the medial subchondral bone decreased (Figures 2B,C). These findings were further confirmed by Western blot analysis, which showed similar trends at the protein level (Figure 2D). In addition, immunofluorescence staining demonstrated increased CD31 expression in the medial subchondral bone compared with the lateral side (Figure 2E).

As for this, we verified the gene expression changes of LEP and LEPR through clinical samples, which were consistent with the trend of the previous bioinformatics analysis results. Meanwhile, increased CD31 expression was observed in the medial subchondral bone, indicating enhanced vascular presence in the lesion area.

### Early OA pathological changes in subchondral bone of mice with LEP and LEPR gene deletion

**Figure 3:**
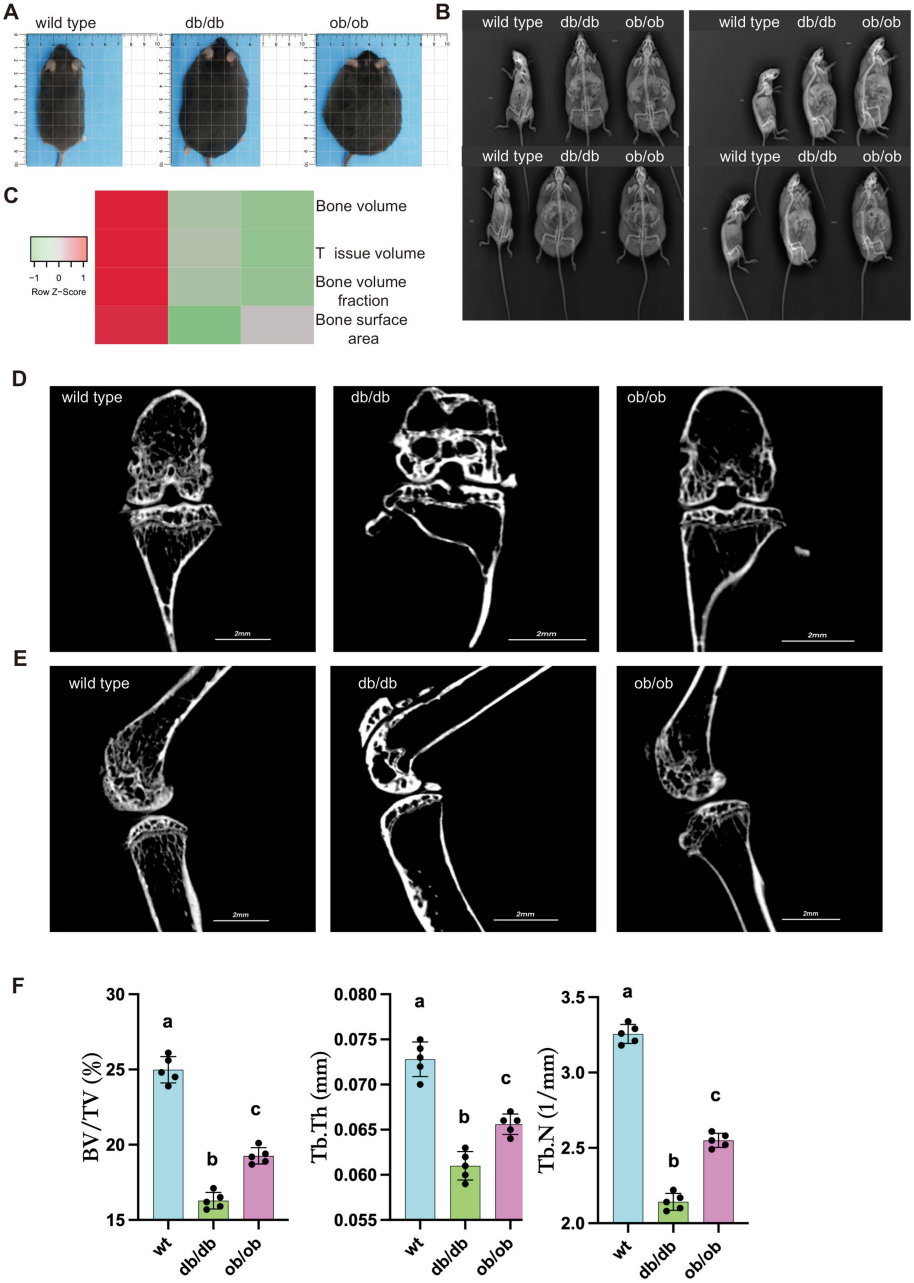
**(A)** General appearance of 6-month-old wild type, db/db, ob/ob mice. **(B)** Front and side X-ray display of bones and joints of 6-month-old wild type, db/db, ob/ob mice. **(C)** Heatmap showing virous parameter of micro-CT scanning in the transgenic mice. **(D)** Representative bone tissue micro-CT scan image from the front view. **(E)** Representative bone tissue micro-CT scan image from the side. **(F)** Quantitative analysis of bone volume fraction (BV/TV), trabecular thickness (Tb.Th), and trabecular number (Tb.N) in WT, db/db, and ob/ob mice. Data are presented as mean ± SD (*n* = 6). Different lowercase letters **(A–C)** indicate significant differences between groups (*p* < 0.05).

To better understand the function of LEP and LEPR genes in subchondral bone, we generated 6–7-week-old wild type mice (db/db background mice, as control group), db/db mice (LEPR homozygous mutation), and ob/ob mice (LEP homozygous mutation). Compared with wild type mice, db/db and ob/ob mice showed strong appetite and slow movements. Grossly, db/db and ob/ob mice were obese and had subcutaneous fat accumulation (Figure 3A). Although no obvious joint deformities or gait abnormalities were observed either in db/db or ob/ob mice (Figure 3B), the bone volume, tissue volume, bone volume fracture and bone surface area are all decreased in the mice with LEP/LEPR homozygous mutation (Figure 3C). Micro CT scanning shows decreased number of trabecular bones in db/db and ob/ob mice (Figures 3D,E) (26, 27). Quantitative micro-CT analysis further demonstrated that BV/TV, trabecular thickness (Tb.Th), and trabecular number (Tb.N) were significantly reduced in both db/db and ob/ob mice compared with wild type controls, with the lowest values observed in db/db mice (Figure 3F).

### Either LEP or LEPR is essential for joint microstructure maintaining

**Figure 4:**
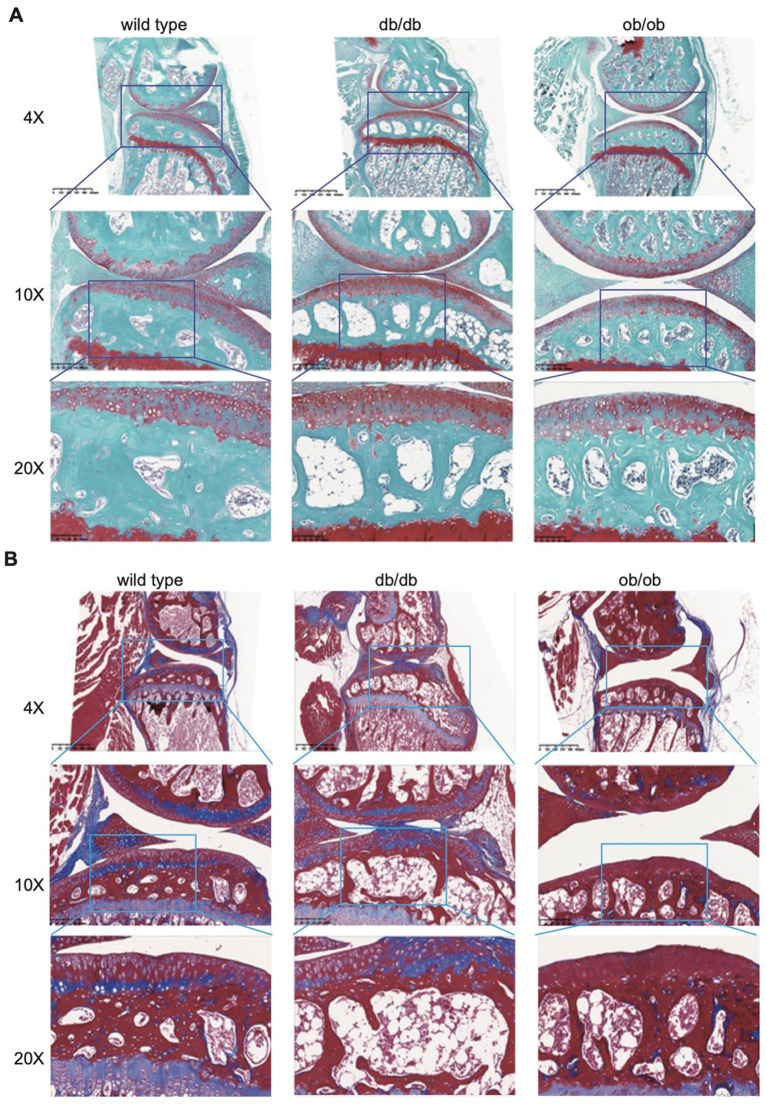
**(A)** Representative safranine O/fast green stain of 6-month-old wild type, db/db, ob/ob mice joint. **(B)** Representative Masson stain of 6-month-old wild type, db/db, ob/ob mice joint.

To ascertain the impact of LEP and LEPR loss on joint microstructure, we fixed, decalcified, and embedded the joints of the sacrificed mice, obtained sagittal sections, and examined the morphology of the articular cartilage using safranin O-fast green staining. Upon examination, the joint surfaces of the three mice exhibited a smooth texture, devoid of fissures or indications of deterioration. Safranin staining revealed varying degrees of loss across each group; however, no significant differences were seen (Figure 4A). We observed a notable increase in fat vacuoles and a reduction in bone mass in the subchondral bone of db/db and ob/ob mice, particularly in db/db mice. This resembles the occurrence of lipid accumulation, cystic alterations, and cavity formation in the subchondral bone on the affected side, frequently reported clinically; nevertheless, the data revealed no indication of harm to the cartilage structure in mice due to lipid accumulation. To enhance comprehension of the development of fat vacuoles beneath the cartilage resulting from the absence of LEP and LEPR genes.

To further investigate the phenomena of collagen, decrease, we employed Sirius red staining. Collagen fibers in the tissue appeared red using a standard optical microscope; however, assessment of different collagen fibers was enhanced using a polarized light microscope. In accordance with the findings from Masson staining, lipid buildup was evident in the subchondral bone of db/db and ob/ob mice, and there was a marked reduction in the red collagen fibers within the cartilage and subchondral bone (Figure 4B). Collagen is a crucial component for preserving bone strength and supplying nutritional support to bones. Collagen can facilitate the deposition of calcium salts in bone tissue. db/db and ob/ob mouse models Sirius red and Masson staining reveal a reduction in subchondral bone collagen, potentially resulting in heightened bone brittleness and precipitating conditions such as osteoporosis and fractures.

**Figure 5:**
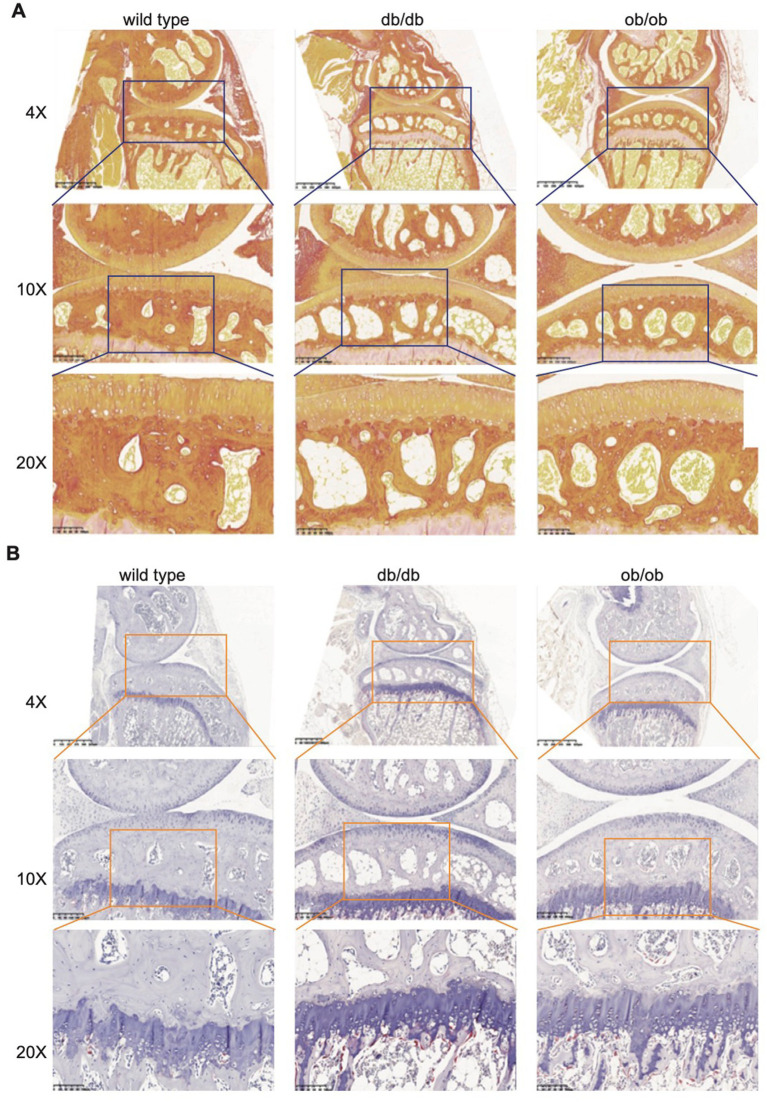
**(A)** Representative Sirius red staining of 6-month-old wild type, db/db, ob/ob mice joint. **(B)** Representative TRAP staining of 6-month-old wild type, db/db, ob/ob mice joint.

In both the early and severe stages of osteoarthritis, the activity of subchondral osteoclasts is elevated, resulting in active subchondral bone remodeling (28, 29). To assess the impact of postnatal loss of LEP and LEPR on osteoclasts in subchondral bone, we employed TRAP staining to examine the distribution and proportion of osteoclasts. Osteoclasts were stained crimson by TRAP and situated in the cytoplasm. The subchondral bone of db/db and ob/ob mice exhibits a limited presence of wine-red patches, however their distribution is not extensive. Nonetheless, a significant quantity of wine-red cells was observed in db/db and ob/ob animals beneath the tidal line (Figure 5A), signifying an increase in osteoclasts in this region and aggressive bone breakdown, which aligns with the characteristics of early osteoarthritis.

The histological staining revealed that the postnatal loss of LEP and LEPR resulted in lipid metabolic abnormalities in subchondral bone, leading to lipid buildup. While this occurrence did not alter the structure of cartilage in its normal form, subsequent analysis revealed a reduction in the amount of subchondral collagen fibers and increased osteoclast activity, aligning with the findings of early clinical osteoarthritis.

Deletion of the LEP and LEPR genes expedites cartilage degradation in murine models.

Cartilage degeneration correlates with atypical subchondral bone remodeling. To ascertain if the alterations in the subchondral bone induced by LEP and LEPR gene deletion impact the chondrocytes in the upper cartilage, we employed toluidine blue to stain the mouse joints. Post-scan analysis revealed that the overall architecture of the cartilage and subchondral bone aligned with the findings from Safranin O-Fast Green staining, indicating an increase in vacuole count within the subchondral bone, while no significant cracks or defects were observed in the cartilage. Notably, the quantity of hypertrophic chondrocytes stained by toluidine blue in the cartilage of db/db and ob/ob mice exhibited a marked increase (Figure 5B).

**Figure 6:**
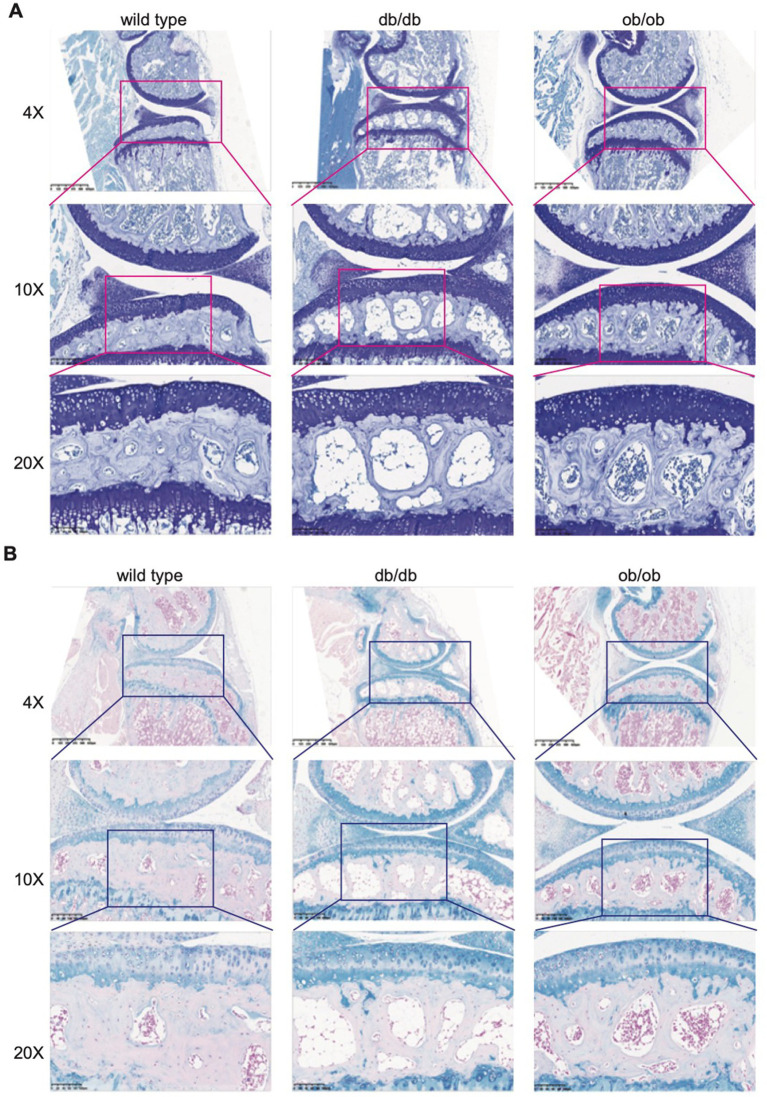
**(A)** Representative toluidine blue staining of 6-month-old wild type, db/db, ob/ob mice joint. **(B)** Representative Alcian Blue staining of 6-month-old wild type, db/db, ob/ob mice joint.

Col-X is a molecular marker expressed during chondrocyte differentiation and hypertrophy, primarily involved in the transformation of chondrocytes into osteoid cells. In the progression of osteoarthritis, chondrocytes progressively deteriorate in both morphology and function, subsequently secreting excessive collagen type X, which exacerbates the structural and functional impairment of cartilage [24]. We employed immunohistochemical labeling to examine the expression of the chondrocyte-specific marker Col-X. In accordance with the findings from toluidine blue staining, the expression of Col-X in the cartilage of db/db and ob/ob mice was markedly elevated (Figure 6A).

To elucidate the impact of chondrocyte hypertrophy resulting from the absence of LEP and LEPR on cartilage components, we employed Alcian blue (A-B) staining to examine alterations in cartilage tissue composition. Alcian blue is a specialized dye for acidic mucopolysaccharides. It causes acidic chondroitin, glycosaminoglycans, and mucopolysaccharides in the tissue to exhibit a blue hue, whereas neutral glycoproteins and collagen display a red hue, so differentiating acidic glycoproteins from neutral glycoproteins. In comparison to the cartilage of wild type mice, the blue coloration of the articular cartilage in db/db and ob/ob mice was markedly more pronounced (Figure 6B), suggesting a large increase in the concentration of acidic glycoproteins. Their anomalous elevation may result in cartilage degradation and exacerbation of the inflammatory response (30, 31). The findings indicate that the postnatal deficiency of LEP and LEPR results in lipid accumulation within the subchondral bone, an elevation in cartilage mast cells, an increase in intracartilage acidic glycoproteins and mucopolysaccharides, alterations in cartilage constituents, and initial indications of cartilage degeneration.

### Elevated H-type blood vessels in the subchondral bone of mice with deletions of the LEP and LEPR genes

**Figure 7:**
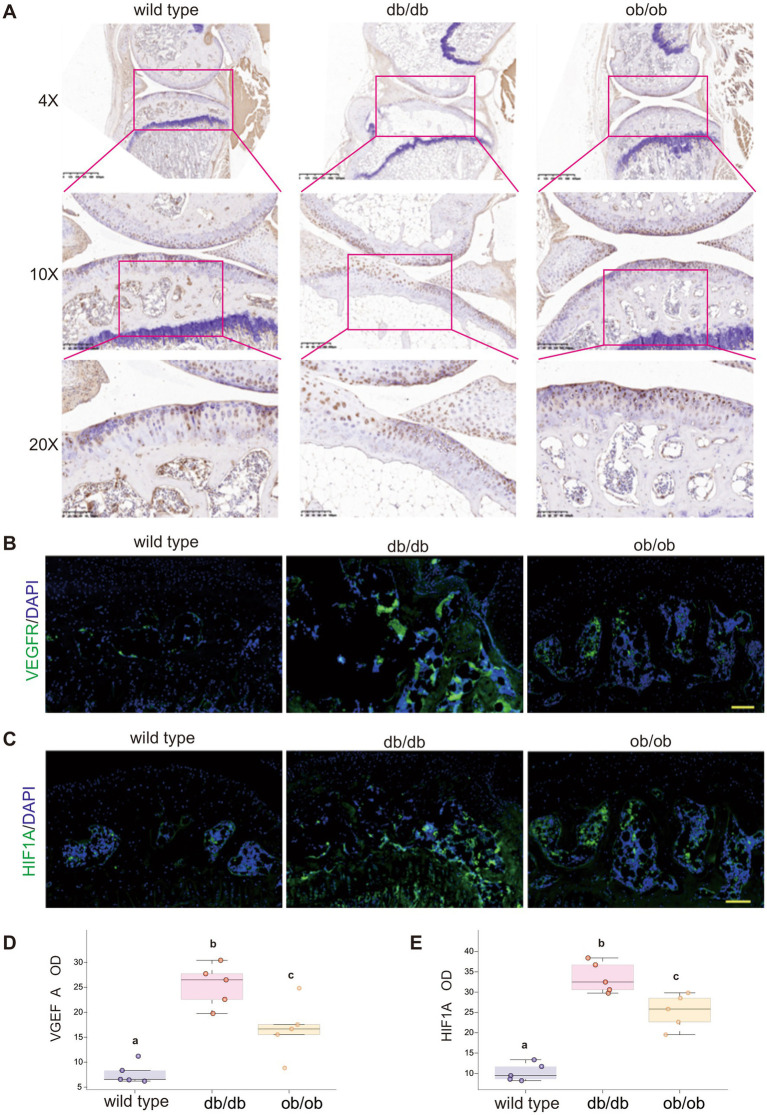
**(A)** The expression of Col-X in the joints of 6-month-old wild type, db/db, ob/ob mice was detected by IHC. **(B)** The expression of VEGFR in the joints of 6-month-old wild type, db/db, ob/ob mice was detected by immunofluorescence. **(C)** The expression of HIF-1α in the joints of 6-month-old wild type, db/db, ob/ob mice was detected by immunofluorescence. **(D)** Quantification analysis of VGEFA optical density. **(E)** Quantification analysis of HIF1A optical density. Different lowercase letters **(A–C)** indicate significant differences between groups (*p* < 0.05).

The density of H-type blood vessels in the subchondral bone correlates with the onset and progression of OA. Abnormal lipid metabolism has been linked to the growth of H-type blood vessels. To further examine whether lipid accumulation in the subchondral bone resulting from the loss of LEP and LEPR genes stimulates the formation of H-type blood vessels, we employed immunofluorescence (IF) and immunohistochemistry (IHC) to detect the angiogenesis markers VEGFR, HIF-1α protein, and the specific marker CD31 protein associated with H-type blood vessels. The elevation of VEGFR enhances the sensitivity of H-type vascular endothelial cells to VEGF signals (32). The green fluorescence of VEGFR surrounding the subchondral bone in db/db mice exhibited a considerable rise, while the subchondral bone in ob/ob mice also shown an upward trend compared to wild type animals (Figure 7A). Hypoxia-inducible factor-1α (HIF-1α) is a transcription factor that is activated in response to hypoxic conditions. HIF-1α has been reported to induce the development of H-type blood arteries. We employed immunofluorescence to ascertain the expression level of HIF-1α in subchondral bone. In accordance with the VEGFR findings, robust green fluorescent protein expression was observed in the subchondral bone of db/db and ob/ob mice, particularly pronounced in the subchondral bone of db/db mice (Figure 7B).

**Figure 8:**
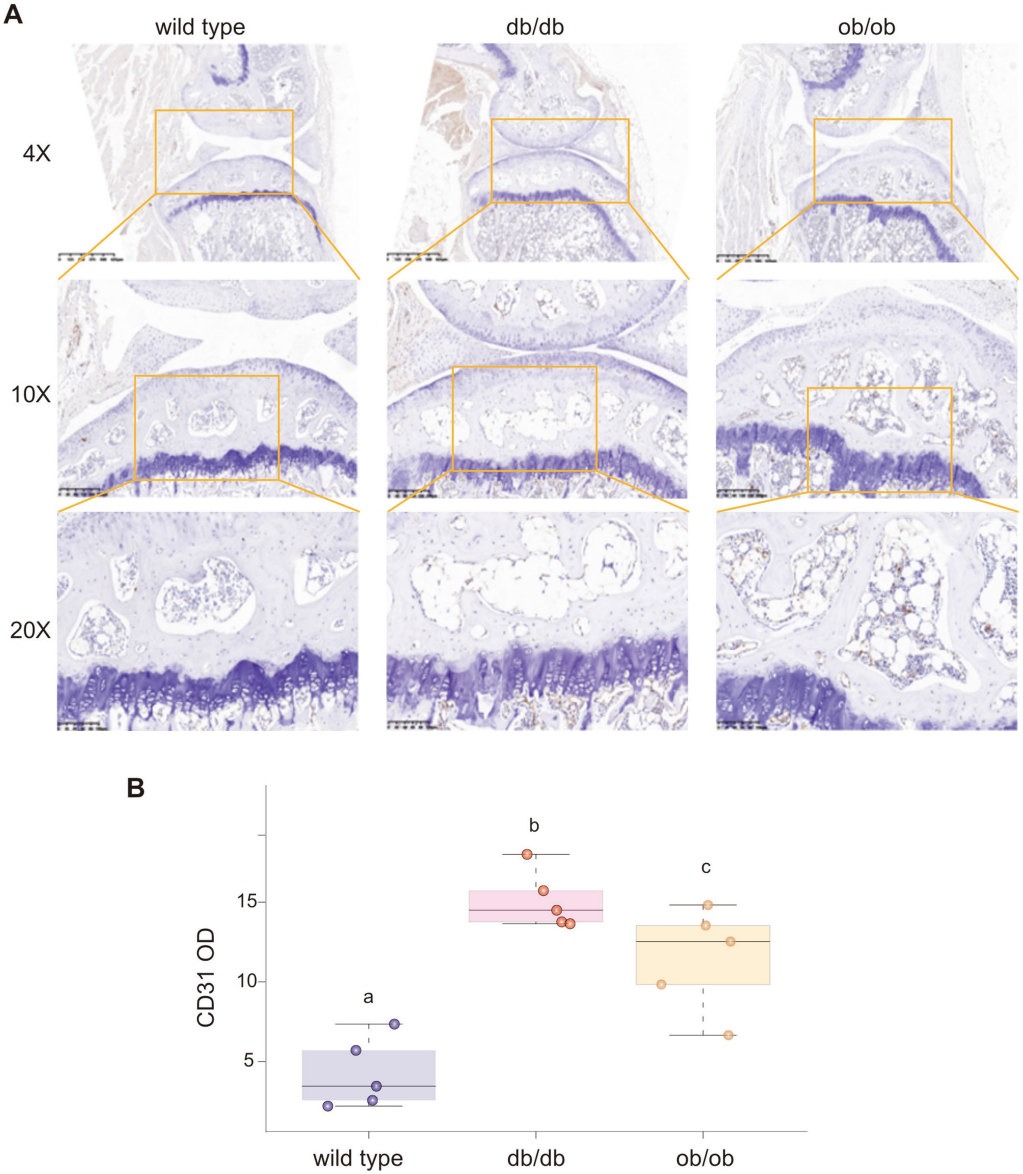
**(A)** The expression of CD31 in the joints of wild type, db/db, ob/ob mice was detected by IHC. **(B)** Quantification analysis of CD31 optical density. Different lowercase letters (a–c) indicate significant differences between groups (*p* < 0.05).

CD31 protein serves as a unique marker for H-type vascular endothelial cells. We employed immunohistochemistry to stain the subchondral bone of mice (Figure 8A). In accordance with the distribution patterns of VEGFR and HIF-1α in the three mouse models, CD31 exhibited elevated expression in the subchondral bone of db/db and ob/ob animals (Figure 8B), indicating an over proliferation of H-type blood vessels in the subchondral bone of these mice (Figures 7C–E).

## Discussion

In order to illustrate the upstream factors regulating H-type vessels, we developed a mouse model with postnatal deletion of LEP and LEPR to assess the impact of LEP and LEPR signaling on the bone architecture and collagen ratio of subchondral bone, as well as on chondrocyte hypertrophy and alterations in intracartilage glycoproteins, which align with the initial modifications in subchondral bone and cartilage observed in human osteoarthritis. These findings emphasize that the focus of this study is on subchondral bone remodeling and its direct association with H-type vessel formation, highlighting the pivotal role of vascular changes in early OA pathology. Simultaneously, we confirmed that the influence of LEP and LEPR on subchondral bone may be mediated through the modulation of lipid metabolism, subsequently affecting H-type blood vessels, so offering valuable insights for the future understanding and prevention of OA.

Recent research increasingly indicates that aberrant lipid metabolism, particularly abnormalities of fatty acid metabolism, may contribute to the onset and progression of OA (33–35). Aberrant lipid metabolism facilitates the onset and advancement of osteoarthritis by multiple pathways, including inflammation, cartilage degradation, subchondral bone sclerosis, and disturbances in chondrocyte anabolism and catabolism (36–38). Lipids are essential constituents of cell membranes and serve as signaling molecules that modulate several cellular activities (39). Aberrant lipid metabolism may result in lipid deposition in joints, causing inflammation and cartilage deterioration. Lipids, including free fatty acids and cholesterol, initiates cartilage deterioration by enhancing the synthesis of inflammatory cytokines and degradative enzymes (40). Lipid metabolism influences the equilibrium between chondrocyte anabolic and catabolic activities. Dysregulation of lipid metabolism may also influence the activity of enzymes critical for collagen and proteoglycan production, which are vital for cartilage integrity (41). Adipose tissue serves as the primary location for lipid metabolism and synthesizes various adipokines, including adiponectin, leptin, and resistin, which influence cartilage metabolism, inflammation, and angiogenesis, so facilitating the onset and advancement of osteoarthritis (42, 43). Lipid metabolism problems concurrently result in an imbalance between lipogenesis and lipolysis (44). Lipids accumulate in the subchondral bone, potentially resulting in subchondral bone sclerosis, a hallmark of osteoarthritis (45). This investigation revealed significant lipid buildup in the subchondral bone of db/db and ob/ob mice. The buildup of lipids diminished the amount of native subchondral bone tissue. Masson staining and Sirius red staining revealed a reduction in the collagen content of the subchondral bone. The reduction in collagen was correlated with a decline in bone mass, resulting in a thinner and more porous subchondral bone plate. It may exhibit increased susceptibility to fracture and separation under circumstances of trauma or severe external force. Concurrently, we also noted that the presence of active osteoclasts in the subchondral bone of ob/ob mice signifies that the subchondral bone undergoes active transformation and exhibits an increase in bone remodeling sites, resembling the characteristics of subchondral bone in both early and severe stages of osteoarthritis. Despite the absence of observable cartilage loss or destruction in mice with postnatal LEP and LEPR impairment, we noted an increase in mast cells and acidic glycoproteins inside the cartilage tissue, suggesting that their cartilage degeneration occurred at a more accelerated rate compared to normal mice. Consequently, it can be inferred that postnatal depletion of LEP and LEPR influences the distribution and trabecular architecture of subchondral bone, and these alterations in subchondral bone subsequently impact chondrocyte degeneration. These results underscore that LEP/LEPR signaling plays a critical regulatory role in subchondral bone remodeling, which in turn modulates H-type angiogenesis and cartilage integrity. This experiment did not explicitly determine the type of collagen in the subchondral bone that was diminished, nor the particular acidic glycoprotein in the cartilage that was reduced, together with the precise impacts on cartilage degeneration, necessitating future investigations for clarification.

Osteogenesis is crucial for preserving the integrity and functionality of the mammalian skeletal system. Osteogenic dysfunction and anomalies can result in numerous bone disorders (46, 47). A subtype of capillaries linked to osteogenesis has recently been identified as type H vasculature, characterized by elevated expression of CD31 and endomucin (Emcn) (48). CD31 was selected as a core marker in this study because it is highly expressed in H-type endothelial cells and directly correlates with osteoprogenitor cell proliferation and subchondral bone remodeling, making it a reliable indicator of H-type vessel activity. Type H vessels are situated adjacent to the metaphyseal growth plate and inside the periosteum and endothelium of the diaphysis. Type H arteries are closely encircled by osteoprogenitor cells that express the transcription factor Osterix, a powerful enhancer of bone production (49, 50). Type H vasculature actively facilitates bone formation by secreting substances that promote the proliferation and differentiation of osteoprogenitor cells (51, 52). LEP/LEPR signaling can influence osteogenesis by regulating osteoblast proliferation, differentiation, and survival, while also modulating osteoclast activity, thereby establishing a mechanistic link between metabolic regulation and H-type angiogenesis in subchondral bone. Hypoxia-inducible factor 1α (HIF-1α), Notch, and VEGFR signaling are molecules significantly produced by type H vascular endothelial cells, which might enhance sensitivity to VEGF, hence promoting angiogenesis (48, 53). Angiogenesis in subchondral bone may significantly contribute to the etiology of osteoarthritis. The clusters of type H arteries surrounding subchondral bone islands in a murine model of osteoarthritis (54). Type H vasculature is elevated in the subchondral bone of osteoarthritis model mice and elderly animals (55, 56). Activation of mTORC1 in cartilage enhances VEGF-A production in articular chondrocytes and facilitates the development of H-type blood vessels in subchondral bone, hence exacerbating osteoarthritis (57). H-type angiogenesis in subchondral bone may be a significant characteristic of early osteoarthritis, and that the quantity of H-type blood vessels signify the extent of subchondral bone proliferation and remodeling (58, 59). In this work, we noted an elevation in the expression of CD31, a definitive marker of H-type blood vessels in the subchondral bone of mice with postnatal deletion of LEP and LEPR. This was accompanied by heightened expression and distribution of VEGFR and HIF-1α, suggesting an increase in the proliferation of H-type blood vessels in the subchondral bone of these mice. This may result from the heightened destructive impact of osteoclasts on subchondral bone, leading to an elevation in the production of substances that promote the formation of H-type blood vessels and augmented bone remodeling. Furthermore, it may also result from direct stimulation following the deletion of LEP and LEPR, or from the growth of H-type blood vessels induced by lipid metabolic problems. The precise mechanism requires additional investigation.

Trauma is one of significant risk factors in the onset of osteoarthritis (60). Articular cartilage exhibiting deterioration demonstrates a sluggish or imbalanced reparative process following damage. This experiment previously demonstrated that mice with postnatal LEP and LEPR impairment exhibited weight growth, increased adiposity, H-type angiogenesis in subchondral bone, and cartilage degradation, all of which are risk factors for OA. Following cartilage damage, malfunction in cartilage repair or aberrant reconstruction of subchondral bone may arise due to an increase in H-type blood vessels in the subchondral bone, exacerbating cartilage degradation. Nonetheless, following the simulation of DMM surgery in the mice, we did not see significant OA cartilage degradation, which contradicted our anticipated outcomes. This investigation is warranted due to the evident obesity in db/db and ob/ob mice, particularly characterized by significant abdominal fat buildup. In a static condition, the interaction between abdominal adipose tissue and the base of the cage alleviates joint stress, rendering the DMM model insignificant in the cartilage-to-cartilage contact; conversely, db/db and ob/ob mice may exhibit reduced physical activity and remain in a static state for extended periods, resulting in a lack of relative mobility among joints. Consequently, we did not include images of mice post-DMM surgery in this study.

In conclusion, experimental confirmation of mice with LEP and LEPR deficiencies confirmed the link between the LEP-LEPR axis and subchondral bone H-type angiogenesis as well as articular cartilage degradation. These findings highlight that LEP/LEPR regulates subchondral bone remodeling through its effects on osteoblasts and osteoclasts, which in turn modulates H-type angiogenesis and contributes to cartilage degeneration, emphasizing the central focus of this study on subchondral bone–vascular interactions in OA progression. The LEP-LEPR axis may regulate H-type angiogenesis and exacerbate articular cartilage degradation.

## Data Availability

The datasets presented in this study can be found in online repositories. The names of the repository/repositories and accession number(s) can be found in the article/Supplementary material.

## Footnotes

1
https://cn.string-db.org

## Supplementary material

SUPPLEMENTARY FIGURE 1 **(A)** 20 sample data before normalized. **(B)** Overview of DEGs. **(C)** Volcano map of DEGs. **(D)** Variation trend map of DEGs. **(E)** Display of the relationship between the main enriched KEGG pathway. **(F)** Hub genes of DEGs (*P* < 0.05, log2 ≥1 or ≤−1) were screened by Cytoscape.
