# Supplementary material for: Subchondral H-type blood vessel formation aggravates articular cartilage degeneration through LEP-LEPR axis
Source: Front Med (Lausanne). 2026 Mar 13;13:1751127. doi: 10.3389/fmed.2026.1751127 (PMC13021791; doi:10.3389/fmed.2026.1751127)
Supplement: Supplementary file 1 [file Image_1.pdf]

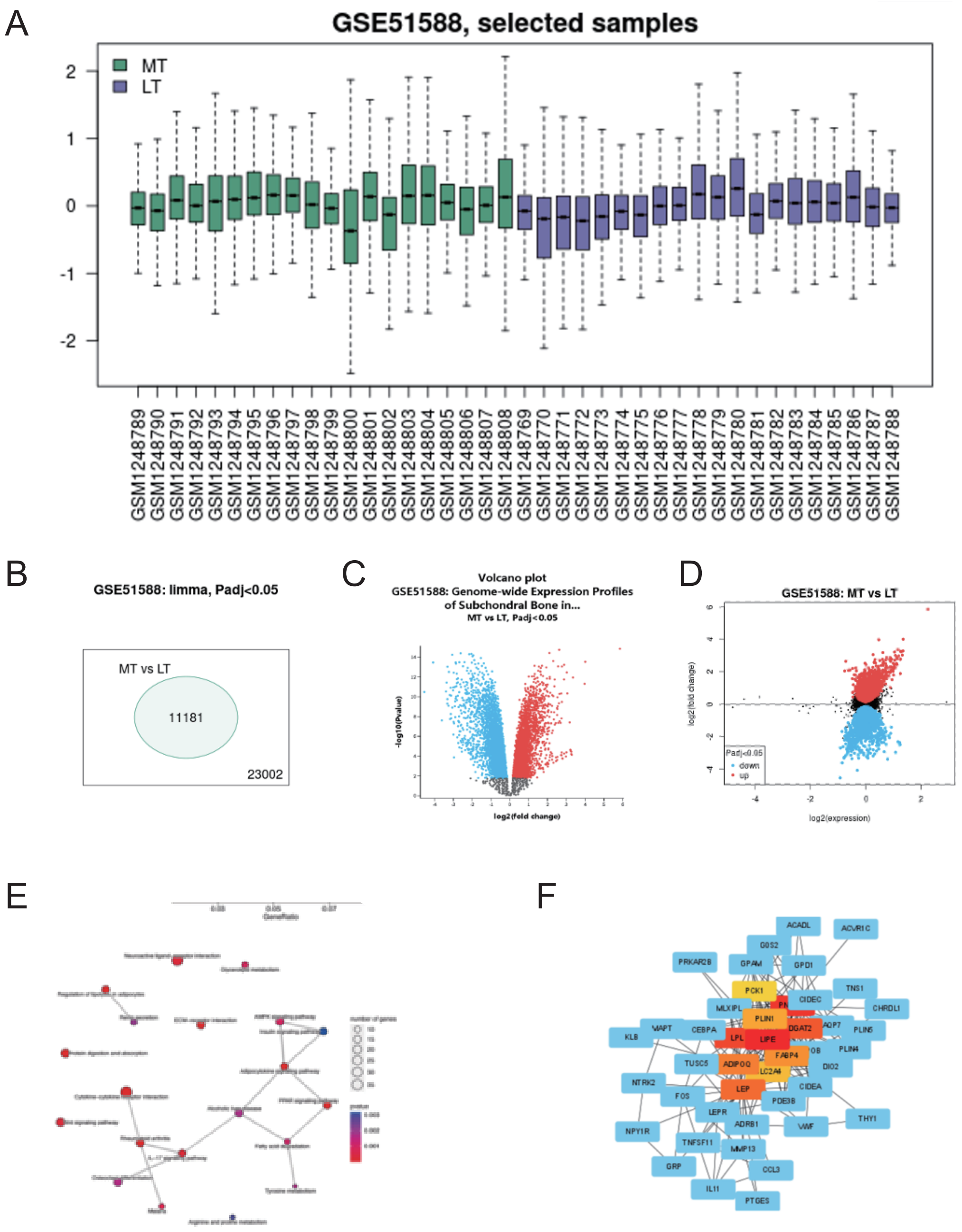
A
GSE51588, selected samples
MT
LT
B
GSE51588: limma, Padj<0.05
MT vs LT
11181
23002
C
Volcano plot
GSE51588: Genome-wide Expression Profiles of Subchondral Bone in...
MT vs LT, Padj<0.05
-log10(Pvalue)
log2(fold change)
D
GSE51588: MT vs LT
log2(fold change)
log2(expression)
Padj<0.05
down
up
E
GeneRatio
number of genes
pvalue
F
ACADL
ACVR1C
G0S2
PRKAR2B
GPAM
GPD1
PCK1
TNS1
CIDEC
CHRDL1
MLXIPL
PLIN1
DGAT2
KLB
MAPT
CEBPA
LPL
LIPE
AQP7
PLIN5
PLIN4
FABP4
TUSC5
ADIPOQ
DIO2
SLC2A4
NTRK2
LEP
CIDEA
FOS
PDE3B
LEPR
THY1
ADRB1
VWF
NPY1R
TNFSF11
MMP13
GRP
CCL3
IL11
PTGES
